# The viscoelasticity of the rubber-ice interface determined by resonance shear measurement: influence of rubber *T*_g_

**DOI:** 10.1080/14686996.2025.2554049

**Published:** 2025-09-01

**Authors:** Michael C. Stevens, Jon Pallbo, Kazue Kurihara, Masashi Mizukami

**Affiliations:** New Industry Creation Hatchery Center (NICHe), Tohoku University, Aobra-ku, Sendai, Japan

**Keywords:** Surface forces, rubber-ice interface, premelting, viscoelasticity, friction, T_g_

## Abstract

We performed resonance shear measurements (RSM) using the low-temperature surface force apparatus (LT-SFA) to investigate how rubber composition influences the viscoelasticity of the rubber-ice interface. RSM data showed quite different behaviours depending on the styrene contents (5, 23 and 45 wt%) of poly(styrene-co-butadiene) rubbers. A mechanical model for RSM was applied to obtain the interface’s viscous (*b*_s_) and elastic (*k*_s_) parameters across a temperature range of *ca*. −20°C to 0°C. All rubber-ice interfaces at a temperature of *ca*. −18° to −10°C showed a significant decrease in viscosity of 1 to 2 orders of magnitude in the maximum compared to the silica-ice interface, presenting properties of the ice premelted layer. This was attributed to the dominant viscoelastic contributions of the rubber with decreasing styrene content, and therefore to the decreasing glass transition temperature (*T*_g_ = –74, −55, and −31℃). The decrease in the viscosity was enhanced more for lower *T*_g_ rubbers. Between −10°C and −5°C, the rubber-ice viscosities converged at a value lower than silica-ice, which was indicative that the interfacial viscoelasticity in this regime was determined by increased contributions from the premelted layer of ice which was probably modulated by polymer-ice interactions. Finally, above −5°C all samples showed a rapid decay in viscosity and elasticity, suggesting that the premelted layer of ice is the main contributor. This study successfully demonstrated that rubber composition could have a profound impact on the viscoelasticity of the rubber-ice interface.

## IMPACT STATEMENT

Resonance shear measurements revealed how rubber composition, thus *T*_g_, affects the viscoelasticity of the rubber-ice premelted layer interfaces, which is important to mediate rubber-ice friction.

## GRAPHICAL ABSTRACT

**Figure:**
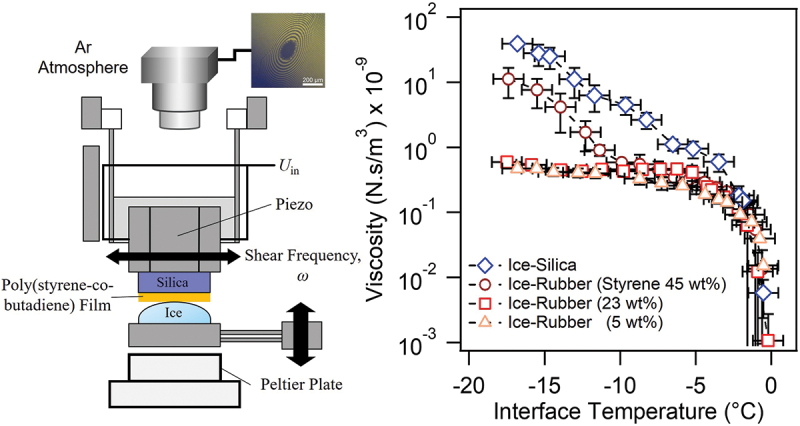

## 1. Introduction

Ice friction plays a pivotal role in a variety of fields including transportation (*e.g*. tire treads), winter sports (*e.g*. ice-skating) and machining (*e.g*. ice breakers) [1–3]. Each application utilises a variety of materials, such as synthetic rubbers and stainless steel, which possess differing properties, including viscoelasticity, surface roughness, and adhesion, all of which can influence friction [4]. With respect to rubber tires, the desired property is high friction to ensure strong grip on road surfaces whilst minimising wear to ensure the safety and longevity of vehicular transport [5]. The behavior of dry rubber friction contacts has therefore been thoroughly investigated by the likes of Schallamach and Grosch, for example, with interfacial properties being evaluated as a function of temperature and velocity [6–8]. These inquiries have suggested that the main contributions arise from rubber viscoelastic deformation, adhesion and wear of the contacting surfaces. Moreover, the addition of water to a rubber surface has been shown to effectively lubricate the contact region, whilst friction is still dominated by the deformation losses of rubber [9]. With respect to ice contact, however, the mechanisms to how rubbers mediate slipperiness at the interface have yet to be fully determined.

Three mechanisms have been proposed to interpret the lubrication of ice surfaces: the formation of a liquid layer through pressure melting and frictional heating, or premelting layer of ice [10–13]. Regarding the first, it has been shown that pressure melting cannot explain ice slipperiness at temperatures below *ca*. −20°C as liquid water cannot coexist with ice in this range and may only contribute at temperatures close to the melting point [12–14]. As a result, the second mechanism of the frictional heating has garnered more support with studies as early as 1939 [15] and others [11,13], addressing the frictional differences between different materials at the ice interface (*i.e*. metal and wood) being explained by such phenomena. Nevertheless, the existence of a premelted layer of ice (hypothesised by Faraday in 1850 due to the observed regelation of ice [16]) has been recently determined by experimental techniques such as X-Ray diffraction and atomic force microscopy [17,18]. Despite this evidence, the contribution of the premelting of ice to lubrication at an ice interface (*e.g*. rubber-ice) has been only suggested [19], and not experimentally demonstrated.

Resonance shear measurements (RSM) [20–22], using the low-temperature surface force apparatus (LT-SFA) [23] based on SFA for opaque samples (twin-path SFA) [24], presents a novel approach to experimentally elucidating the viscoelastic properties of ice-solid interfaces [25–27]. The characteristics can be measured as a function of temperature, sliding velocity, and pressure at small junctions, typically of the order of *ca*. 10,000 µm^2^, thereby bridging the gap between the nanoscopic and macroscopic phenomena. Prior studies using RSM showed the viscosity of the premelted ice layer being 3–5 orders of magnitude larger than that of bulk water (assuming an interfacial layer thickness, *h*, of 1 nm), and exhibited a decay in the viscosity with increasing temperature above −18°C [25]. These findings were then applied to a vulcanised rubber in contact with ice and concluded that the interface’s friction was solely dependent on the rubber’s viscoelastic properties at temperatures below −5°C, then on the premelting properties of ice above −5°C [27].

Considering these results, it is important to study how rubber viscoelasticity, which could be changed by varying the rubber composition, affects the ice-rubber interfacial behaviour. In this study, we aimed to systematically vary the styrene content (and therefore glass transition temperature, *T*_g_) of rubber samples, poly(styrene-cobutadiene), and measure the viscoelasticity of the rubber-ice interface. This was performed by RSM across a temperature range of approximately −20°C to 0°C. Our results illustrated the extent to which rubber and ice contribute to interfacial properties and therefore provide a foundation for understanding the mechanisms of slip at the ice interface.

## 2. Experimental

### 2.1. Materials

**Figure 1.**
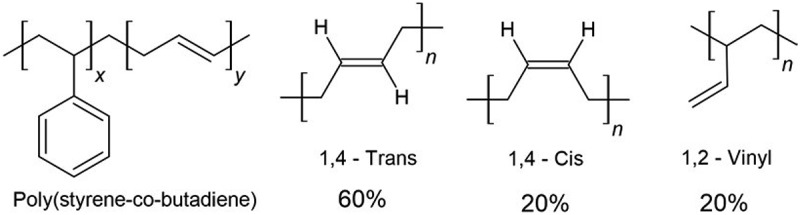
ChemSketch [28] illustration of poly(styrene-co-butadiene) and the butadiene isomer composition.

Three poly(styrene-co-butadiene) polymers comprising 5, 23 and 45 wt% of styrene (Figure 1, Scientific Polymer Products, Inc) were used as supplied. Their *T*_g_ values were determined by using differential scanning calorimetry (RIGAKU DSC8230L) to be −74°C, −55°C and −31°C, respectively (*cf*. supplementary information, Figure S1). Polymer solutions (*c* = 40, 45 or 50 mg/mL) were made in toluene (99.5%, FUJIFILM Wako Pure Chemical Co. Ltd.), and used to spin-coat silica and silicon substrates. Ice was formed using ultrapure water (18.2 MΩ, < 3ppb organics) produced using a Barnstead,

NANOpure Diamond equipped with a double distillation system. Mica sheets (M.

Watanabe & Co.,Ltd.) were used to prepare templates for preparing ice.

### 2.2. Reflectometer

The thickness of polymer films was determined by reflectometry. Silicon wafer (Nilaco Corporation, > 1000 Ω cm) was cut into ~ 2×2 cm squares and cleaned with a piranha solution. The rubbers, dissolved in toluene (*c* = 40, 45 or 50 mg/mL), were spin coated (Mikasa Opticoat, MS-A100) onto silicon wafer pieces with two consecutive cycles (1000 rpm, 120 s and 2000 rpm, 3 s, respectively). Samples were subsequently vacuum dried for 1 h in a desiccator. Once dried, the samples were positioned with an incidence angle (*θ*_i_) of 0° below a UV/Vis light source (Photal, MC-2530). Spectral reflectance was measured as a function of wavelength (*λ*) between the range of 380 and 780 nm using a Photal MCPD-7000 photo detector. An uncoated silicon wafer acted as a calibration sample to remove the contributions from Si/SiO_2_. The film thickness was estimated with the filmetrics spectral reflectance calculator [29] using a three-layer model, Air-Styrene-Si, and the thickness parameters were manually adjusted until the wave periodicity was matched (*cf*. Figure S2).

### 2.3. Low-temperature surface force apparatus (LT-SFA)

Silica lenses (radius of curvature, *R*_si_ = 20 mm) were cleaned in piranha solution to remove the presence of any contaminants. Polymer solutions (*c* = 40, 45 or 50 mg/mL) were spin-coated onto the silica lenses and vacuum dried using the same settings discussed prior (*cf*. section 2.2). A reference silica lens was plasma treated under a water/Ar atmosphere (80 Pa/0.6 Torr) using a Samco, BP-1, 20 W, 13.56 MHz radio frequency plasma source for 30 minutes to generate a clean, hydroxylated silica substrate prior to every experiment. The upper sample surface (*i.e*. the reference silica lens or spin-coated rubber film) was mounted to a piezoelectric tube suspended by a pair of vertical leaf springs, whereas the lower surface of ice was grown under an inert atmosphere of Ar (flow rate ≈1.0 L/min) using a procedure previously reported in detail by Florian *et al.* [23]. The ice was connected to horizontal leaf springs (spring constant ≈238 N/m) to measure the applied normal load (*L*) between surfaces.

**Figure 2.**
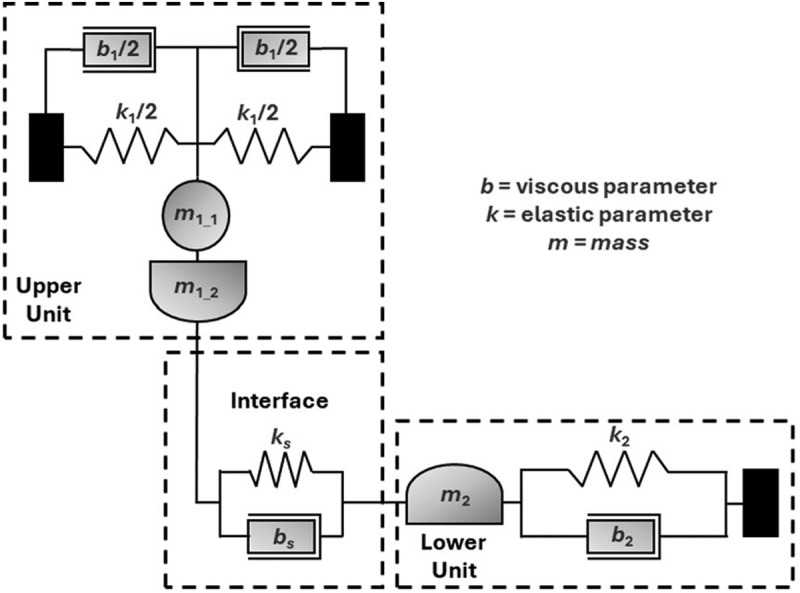
Mechanical model of the LT-SFA [25].

**Figure 3.**
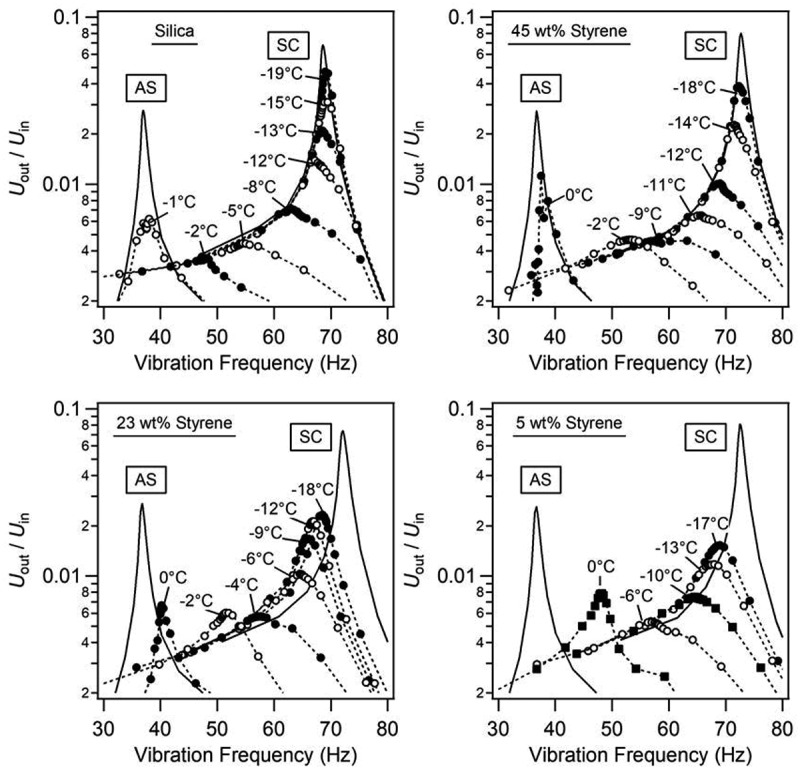
Resonance curves (*U*_out_/*U*_in_ vs vibration frequency) as a function of temperature for silica-ice and each polymer species deposited at concentration, *c* = 50 mg/mL. Each graph is one sample measurement.

A known input amplitude (*U*_in_ = 10 V) of varied frequency (*ω*) was applied to the upper unit’s piezoelectric tube to produce a sinusoidal wave. The deflection of the connected vertical leaf springs was monitored using a capacitance probe to provide the output amplitude (*U*_out_) at incremental changes in *ω*. *U*_out_/*U*_in_ was plotted as a function of *ω* to produce a resonance curve (*cf.* Figure 3). Two reference curves were formed by measuring the rubber-coated silica out-of-contact (AS: air separation) and the bare, plasma treated silica lens in-contact with the mica template (SC: solid contact, *L* = 19 ± 0.5 mN). These controls enabled determination of the viscoelastic parameters of the upper (*b*_1_ and *k*_1_) and lower units (*b*_2_ and *k*_2_), respectively (*cf.* Figure 2). *m*_1_1_, *m*_1_2_, *m*_2_ correspond to the effective masses of the upper unit (excluding upper substrate), upper substrate and lower unit [25].

The AS peak (*ω*_AS_ ≈37 ± 0.1 Hz) and SC peak (*ω*_SC_ ≈71 ± 2 Hz) represent the limits that the silica-ice and rubber-ice resonance curves will shift between depending on the interface’s viscoelastic properties (*b*_s_ and *k*_s_). A decrease in the amplitude and frequency of the resonance curves (Figure 3) corresponds to a decrease in the *b*_s_ and *k*_s_ parameters, respectively. To measure the rubber interface, the mica template (used to form a smooth, curved ice surface) was carefully peeled from the ice surface and the upper unit’s reference silica lens was replaced with a rubber film. At an interface temperature of *T*_s_ ≈ −20°C, a normal load of 19 ± 0.5 mN was applied between the rubber and ice. The temperature was then incrementally increased in step sizes of roughly 1–2°C, performing a scan at each point. Furthermore, an optical microscope positioned above the SFA was used to take images of the surface’s apparent contact area (*A*) as a function of interface temperature, *T*_s_ (*cf*. Figure S3).

The output resonance curves were modelled assuming the interface possesses mixed contributions (monolayer) from the rubber and ice. A Kelvin-Voigt model (as previously reported using RSM [22,26]) was used to extract the viscoelastic parameters of the interface. The equation to fit the resonance curves (*U*_out_/*U*_in_ vs *ω*) is shown as follows, where *C* is an intensity parameter that accounts for the effective transfer constant of piezo bending and the sensitivity of the capacitance probe:

(1) $$\frac{U_{\mathrm{out}}}{U_{\mathrm{in}}}=C\sqrt{\frac{\left(d+e\right)}{\left(f+g\right)}}$$

where

(2) $$d=\{m_{1\_2}m_{2}\omega^{4}-[(m_{1\_2}k_{2})+b_{1}b_{2}+(m_{1\_2}+m_{2})k_{s}]\omega^{2}+k_{s}k_{2}{\}}^{2}$$

(3) $$\;\;e=\{-[m_{1\_2}b_{2}+(m_{1\_2}+m_{2})b_{s}]\omega^{3}+[k_{2}b_{2}+k_{2}b_{s}]\omega {\}}^{3}$$

(4) $$f=[(m_{1\_1}+m_{1\_2})(m_{2}\omega^{4}-k_{2})+m_{2}k_{1}+b_{1}b_{2}+(b_{1}+b_{2})b_{s}+(m_{1\_1}+m_{1\_2}+m_{2})k_{s}{]}^{2}$$

(5) $$g=\{-[m_{2}b_{1}+(m_{1\_1}+m_{1\_2})b_{2}+(m_{1\_1}+m_{1\_2}+m_{2})b_{S}]\omega^{3}-[k_{1}b_{2}+k_{2}b_{1}+(b_{1}+b_{2})k_{S}+(k_{1}+k_{2})b_{S}]\omega {\}}^{2}$$

Finally, the extracted *b*_s_ and *k*_s_ were normalized to the apparent contact area, *A*, using images taken at their corresponding temperatures to account for ice sublimation overtime. The viscous parameter, *b*_s_, normalized to contact area (*A*) is the equivalent of the effective viscosity (*b*_s_/*A≡ η/h*), where *η* is viscosity and *h* is the interfacial layer thickness [27]. To smooth the data for the styrene content study (performed at *c* = 50 mg/mL), the viscoelastic parameters for each sample at the ice interface were averaged across three experiments and the errors in *b*_s_/*A* and *k*_s_/*A* are the standard deviations of these values (*cf*. Figure 6). The film thickness study of the 5 wt% copolymer was performed as a single measurement for each thickness samples (*cf*. Figure 5).

### 2.4. Confocal laser scanning microscopy

Rubber samples deposited on silica lenses were measured using a Shimadzu, SFT-3500 and Olympus OLS3000 at 100x magnification to image a 128 × 96 µm area. Using confocal microscopy, a 3D height profile of the silica lens and spin-coated rubber films was taken. Collected 3D images were processed using Gwyddion [30] (*cf*. supplementary information) and 2D/1D surface roughness analysis was performed (see Table S2, Figure S4).

## 3. Results and discussion

### 3.1. Resonance shear measurement on ice-rubber interfaces

First, we examined the resonance curves and observed distinct changes in amplitude and frequency between different rubber compositions (Figure 3). Silica-ice, as a reference system, showed no shift in peak frequency relative to the solid contact peak (SC) at temperatures below −12°C. On the other hand, the peak amplitude, corresponding to the intensity of friction in this case, constantly decayed with increasing temperature due to a decrease in the viscous parameter.

The 45 wt% styrene rubber-ice system at *ca*. −17°C showed the resonance peak identical in frequency and smaller in amplitude compared to the silica-ice, and shifted to lower frequency and decreased in amplitude with increasing temperature. This indicated their friction was smaller than the silica-ice case. Other compositions of 23 and 5 wt% showed quite different behaviour. The peaks shifted to lower frequencies even at low temperature, and their amplitudes were much less than the previous two cases, indicating their much smaller friction compared to silica and 45 wt% rubber due to low interfacial viscosity and elasticity. These results demonstrated clearly that the rubber mechanical property is an important factor in determining their friction with ice. To understand how friction was modulated by both ice and rubber, we analyzed RSM data in the following sections.

### 3.2. Temperature dependence of the Si-ice interface viscoelasticity

**Figure 4.**
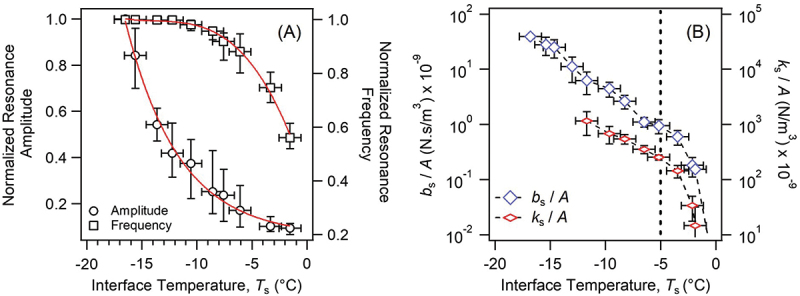
(A) The normalized peak amplitude and frequency of the resonance curves (*cf.* Figure 3) plotted as a function of *T*_s_. The results were averaged across three samples. The red line acts as a guide for the eye. (B) The *b*_s_ and *k*_s_ of the silica-ice interface as a function of *T*_s_ normalized to the apparent contact area, *A*, and averaged across three samples.

The silica-ice interface across a range of temperatures (−18 to 0°C) was measured to demonstrate the premelted ice layer’s viscoelasticity since deformation of silica should be negligible as shown by the previous study [25]. Figure 4A shows changes in the frequency and the amplitude of the resonance peaks. These were normalized to their corresponding values at the lowest temperature to illustrate their relative change as the temperature was increased. The minor shift in the resonance frequency below *ca*. −12°C indicated practically no measurable change in *k*_s_, therefore, the data was modelled to obtain the *b*_*s*_ values assuming there was no deformation of the premelted layer of ice (*k*_s_ = 0). Above −12°C, we could fit the curves to calculate both the *b*_s_ and *k*_s_ values as shown in Figure 4B. Constant decrease in the *b*_s_ value indicated the presence of the premelted layer until −5°C due to increasing temperature likely leading to more disordered surface water molecules under confinement, whilst above −5°C more liquidlike water has formed. The premelting behavior observed in this study may not be identical for all temperature ranges because many different morphologies are found for ice, which could cause different behaviors.

### 3.3. Influence of film thickness on viscoelasticity of the rubber-ice interface

We prepared rubber films on silica by deposition (5 wt% styrene, *c* = 40, 45 and 50 mg/mL) with variable film thicknesses of *t* = 1–2 µm (*cf*. Table S1) to confirm that the viscoelastic properties of the rubber-ice interface were independent of the thickness.

The effective contact area (*A*) of 5 wt% styrene copolymer decreased from ~ 31,000 µm^2^ (*t ≈ 1*.98 µm, *c* = 50 mg/mL) to ~ 15,000 µm^2^ (*t ≈ 1*.35 µm, *c* = 40 mg/mL), indicating that the silica substrate prevented compression in the direction of the applied load.

**Figure 5.**
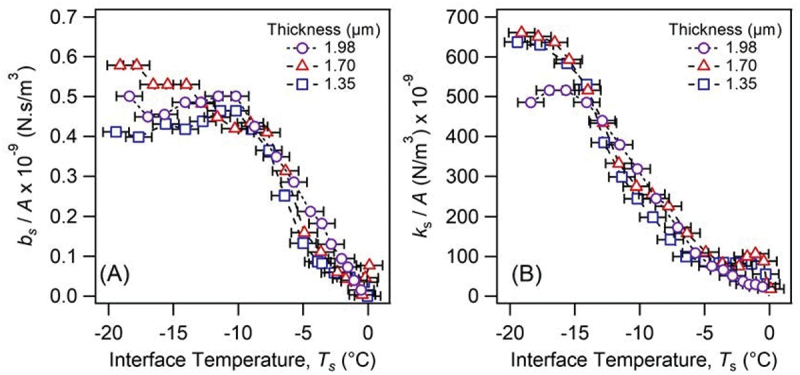
Comparison of (A) *b*_s_/*A* and (B) *k*_s_/*A*, as a function of temperature at the rubber-ice interface for the 5 wt% copolymer at three thicknesses (*t* ≈ 1.98 1.70 and 1.35 µm).

Figure 5 compares the normalized viscoelastic parameters (*b*_s_/*A* and *k*_s_/*A*) of the rubber-ice interface shown as a function of temperature with varied film thickness.

There was no observable difference between the three film thicknesses for the viscoelastic parameters within temperature ranges of *ca*. −10 to 0°C for *b*_s_ and −15 to 0°C for *k*_s_. This indicates that the contact pressure of each film (0.57, 0.95 and 1.27 MPa for *t* = 1.98, 1.70 and 1.35 µm, respectively) has negligible influence on the effective viscosity of the rubber-ice interface. This was previously reported for the silica-ice interface by Lecadre *et al.* [25], in which the result at an applied pressure of 20 MPa was negligibly different from the one at 1 MPa.

However, temperatures below −10°C and −15°C appeared to show a larger variance in the viscous and the elastic parameters, respectively. It has been previously suggested that the viscoelastic properties of rubber dominate the ice interface at lower temperatures [27], therefore, it is likely this variance that was caused by local morphological differences in the rubber film. Confirmation of the film thickness independence for the rubber-ice interface enabled comparisons to be made with the other copolymers 23 wt% (*t* ≈1.54 µm) and 45 wt% styrene (*t* ≈1.25 µm) of similar film thicknesses.

### 3.4. Viscoelasticity of the rubber-ice interface at various styrene contents

**Figure 6.**
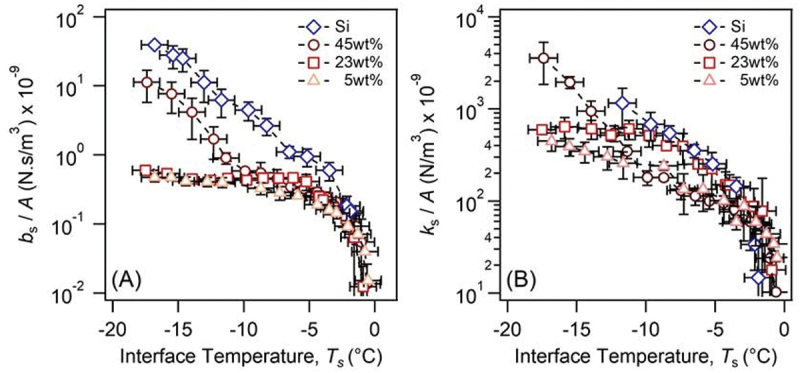
(A) *b*_s_/*A* as a function of temperature at the rubber-ice interface for the 5, 23 and 45 wt% copolymers. (B) *k*_s_/*A* as a function of temperature at the rubber-ice interface.

To investigate how rubber composition can influence the viscoelasticity of the rubber-ice interfacial layer, we studied three poly (styrene-co-butadiene) with different styrene contents and compared with the silica-ice interface. Figure 6 compares the viscosity and elasticity for each rubber film at the ice interface as a function of temperature. They were averaged across multiple experiments (Figures S5-S8) and showed significant differences not only between silica and rubber cases but between different styrene content rubbers. Silica-ice, as previously discussed, exhibits two distinct regions: an exponential decay in viscosity below −5°C, and a much more significant rate of decay above. All the rubber samples (Figure 6A), however, show unprecedented changes in the interfacial layer’s viscosity, with three distinct regions manifesting in the temperature ranges of *ca*. −18°C to −10°C, −10°C to −5°C and −5°C to 0°C.

The low-temperature regime, −18 to −10°C, showed a large decrease in *b*_s_*/A* for all rubber samples by 1 to 2 orders of magnitude when compared to the value for silica.

Regarding the differences between the rubbers at −18°C, we observed the *b*_s_ value of the 45 wt% styrene was one order of magnitude greater than the values for the 23 and 5 wt% samples, among which the *b*_s_ of the 23 wt% was larger than 5 wt%. These values gradually decreased with increasing temperature and converged at −10°C. The *k*_s_ value in this temperature range was in the order of 45 wt% > 23 wt% > 5 wt% and was significant at −18°C.

In the intermediate regime, −10 to −5°C, the change in *b*_s_*/A* was different depending on the styrene content: the value constantly decreased for the 45 wt% with increasing temperature; slightly increased for the 23 wt%; more gradually decreased for the 5 wt%, and in all cases the values were significantly smaller than one of the silica. On the other hand, *k*_s_*/A* showed the 45 wt% and 5 wt% similarly decreasing with increasing temperature at values lower than silica, whereas 23 wt% followed the same tendencies as silica.

Finally, the last region above −5°C shows a rapid decay in *b*_s_*/A* and *k*_s_*/A* for both rubber-ice and silica-ice. This was previously attributed to the decrease in the viscoelasticity of the premelted layer of ice [25].

Higher styrene content for poly(styrene-co-butadiene) polymers has been shown to increase *T*_g_ and possess a larger elastic modulus due to increased monomer rigidity [31]. Here, the 45 wt% (*T*_g_ ≈ −31°C) has a *T*_g_ relatively close to the temperature ranges we investigated, as a result, the rubber film should have restricted polymer mobility and deformability as opposed to the ‘softer’ 23 wt% (*T*_g_ ≈ −54°C) and 5 wt% (*T*_g_ ≈ −74°C) copolymers. This decrease in viscoelasticity should therefore resemble silica less, and hence we observed a smaller viscosity between −18 and −10°C. Figure 6B supported this argument, in which *k*_s_*/A* below −10°C showed a distinct decrease with decreasing styrene content. The silica-ice interface’s elasticity, however, was absent (*k*_s_ = 0) in this region, suggesting the glassy silica and the premelted layer of ice are not elastically deforming ( <−12°C), indicating the viscoelastic deformation of the rubber dominates this regime.

The convergence in *b*_s_*/A* at −10°C was not interpretable by simply considering *T*_g_, and possibly indicative of a collective property between rubbers such as polymer-ice interactions and enhanced ice viscoelastic contributions. First, we considered the surface roughness contribution, which was determined using confocal scanning laser microscopy across an apparent contact area of *ca*. 12000 µm^2^ (for details, see the supplement and Table S2). The averaged surface roughness, *S*_a_, showed the presence of features that were *ca*. 50–80 nm and much greater than silica (a roughness of 9 nm for the same area). Such features, despite having low frequency, could account for the differences in *k*_s_*/A* observed here if the indentation depth was comparable with this roughness value. However, the indentation depths [32] estimated for the rubber-ice interfaces (*cf*. Table S3) assuming elastic deformation was approximately 5–20 times larger, implying that a load of 19 mN should mitigate the impact on viscoelasticity. The polymer-ice interactions or the ice’s viscoelastic properties are thus the main contributors to the reduced rubber-ice viscosity in this region, which is supported by the presence of an elastic parameter for the premelted ice layer (Figure 6B). Future investigations isolating the rubber and ice contributions are necessary to elucidate the mixed phenomena by modifying the model applied here.

## 4. Conclusion

We performed resonance shear measurements using the low-temperature surface forces apparatus to evaluate the viscoelastic properties of the rubber-ice interface by varying the styrene content of a model rubber, poly(styrene-co-butadiene), and comparing their rubber-ice viscoelasticity with the premelted layer of ice.

We observed different resonance curves depending on the compositions and analyzed them using the mechanical model including viscous (*b*_s_) and elastic (*k*_s_) parameters of the interface. We confirmed that the viscoelastic parameters normalized by the apparent contact area (*A*) showed no dependency on the film thickness within the range of this study, *i.e*. 1–2 µm, and enabled comparisons between different rubber compositions of similar film thicknesses.

At temperatures below −10°C, the rubber-ice interface showed an unprecedented decrease in viscosity, *b*_s_/*A*, for all rubber compositions in comparison to silica-ice. The decrease was larger with the lower styrene content and can be expressed in the order of Si > 45 wt% > 23 wt% > 5 wt%. This was attributed to the difference in glass transition temperature (*T*_g_), where a greater styrene content, and thus higher *T*_g_, tended towards silica-ice behaviour due to an increase in rubber viscoelasticity.

Between −10°C and −5°C, the rubber-ice viscosities converged at a value lower than silica-ice, which was indicative that the interfacial viscoelasticity in this regime was determined by increased contributions from the premelted layer of ice probably modulated by polymer-ice interactions. Finally, above −5°C all samples showed a rapid decay in both *b*_s_/*A* and *k*_s_/*A*, suggesting that the premelted layer of ice is the main contributor.

We successfully showed that rubber composition can have a profound impact on the viscoelasticity of the rubber-ice interface. This study demonstrated that optimization of the contact area and shape should be considered more important in industrial applications such as designing shoes and tires, and rubber formulation should be better adjusted by considering different temperature ranges. To better elucidate the mechanism of how the rubber composition modifies the interfacial properties, it is necessary to isolate the rubber and ice contributions, and therefore, identify the extent to which polymer-ice interactions or premelted ice contributes to the interfacial properties.

This is under our current investigation.

## Supplementary Material

Supplemental Material
