# Supplementary material for: The viscoelasticity of the rubber-ice interface determined by resonance shear measurement: influence of rubber Tg
Source: Sci Technol Adv Mater. 2025 Sep 1;26(1):2554049. doi: 10.1080/14686996.2025.2554049 (PMC12481528; doi:10.1080/14686996.2025.2554049)
Supplement: Supplemental Material [file TSTA_A_2554049_SM9827.docx]

**Supplementary Information**

**The viscoelasticity of the rubber-ice interface determined by resonance**

**shear measurement: influence of rubber *T*_g_**

Michael C. Stevens, Jon Pallbo^#^, Kazue Kurihara*, Masashi Mizukami*

*New Industry Creation Hatchery Center (NICHe), Tohoku University, Aoba-ku, Sendai 980-8577, Japan*

*Correspondences:

KK: kazue.kurihara.b7@tohoku.ac.jp

MM: masashi.mizukami.e7@tohoku.ac.jp

*#Current address: Lund University, Naturvetarvägen 22, Lund, Sweden*

**1 Rubber Characterisation**

# 1.1 Differential Scanning Calorimetry (DSC)


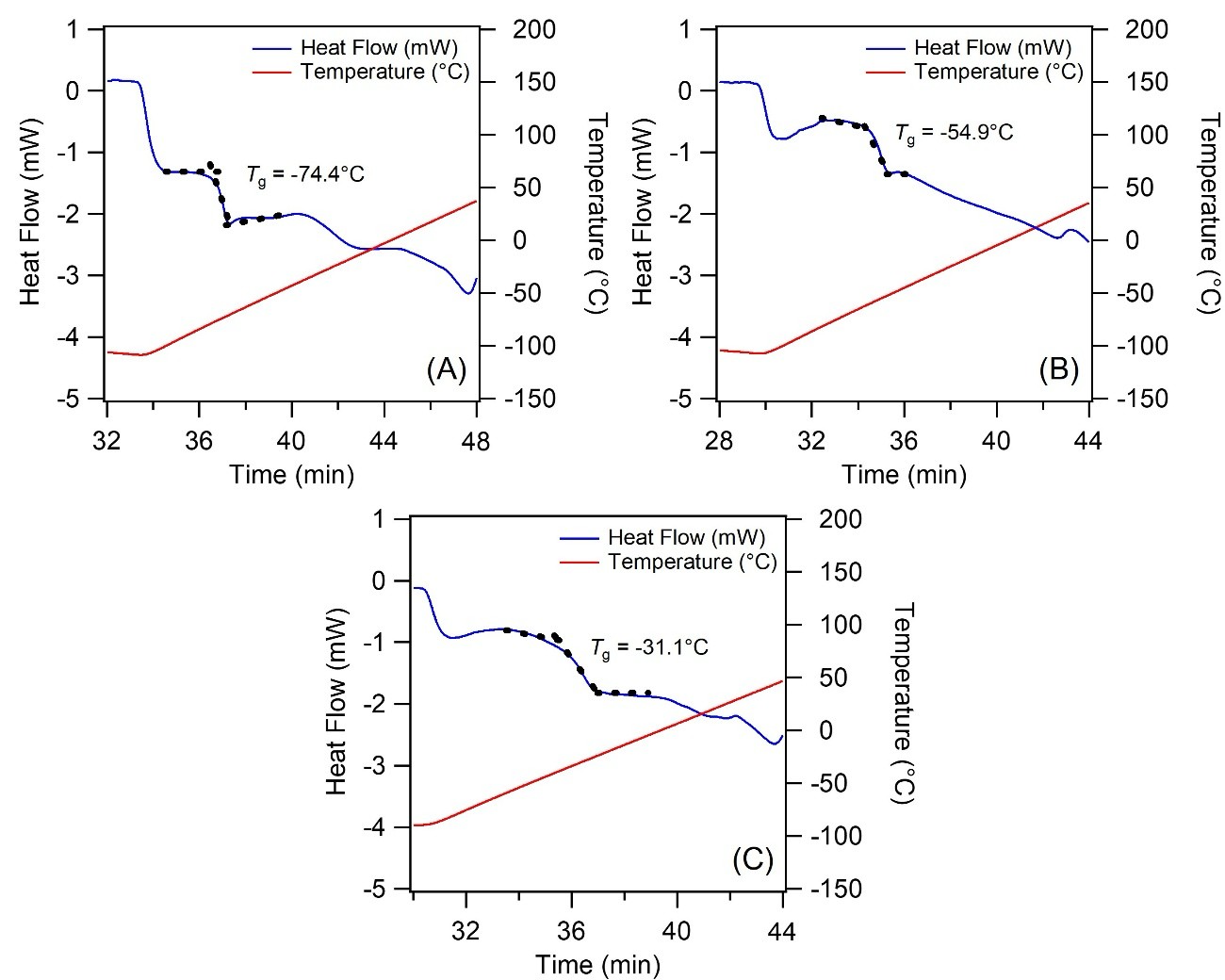


***Figure S1.*** *Heat flow and temperature as a function of time: (A) 5 wt% (B) 23 wt% (C) 45 wt% styrene copolymers.*

# 1.2 Reflectometer


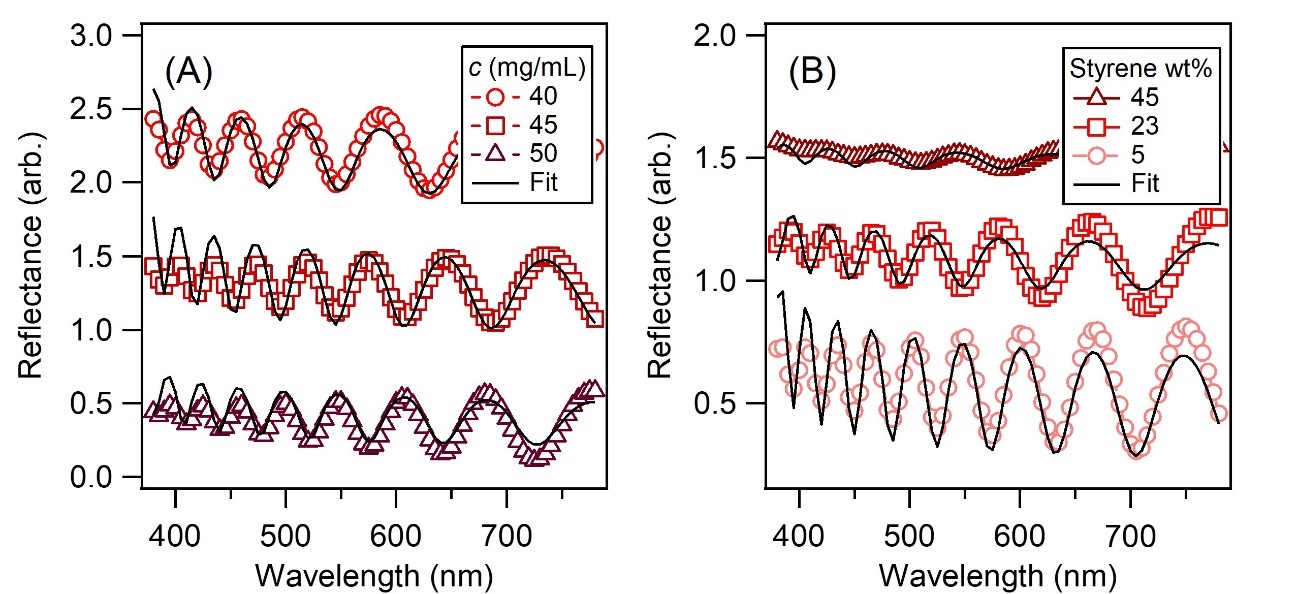


***Figure S2.*** *(A) Reflectometer measurements illustrating the change with concentration, and their corresponding model fits for the 5wt% styrene rubber film. Periodicity of the model was matched, and amplitude was scaled arbitrarily. (B) Reflectometer fits for the varied styrene wt% copolymers (5, 23 and 45wt%).*

# 1.3 Optical Microscope


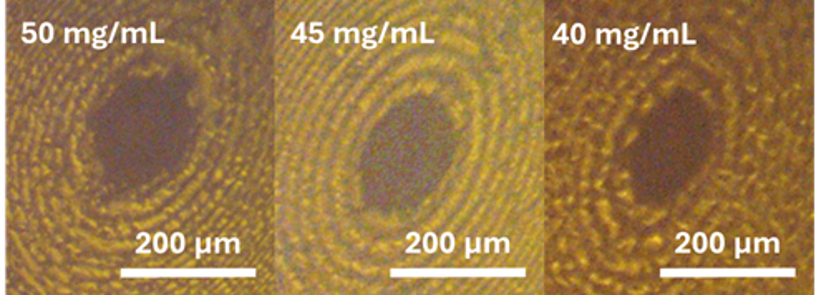


***Figure S3.*** *Example optical microscope contact area images for the 5 wt% styrene rubber-ice interfaces as a function of deposition concentration.*

# 1.4 Film Thickness vs Contact Area

***Table S1.*** *Film thickness measured using the reflectometer for each spin-coated rubber film on a silica len,s and the rubber-ice / silica-ice interface apparent contact area.*

| **Polymer (wt%)** | **Deposition**  **Concentration**  **(mg/mL)** | **Film**  **Thickness, *t***  **(µm)** | **Averaged Apparent**  **Contact Area, *A* (µm^2^)** |
| --- | --- | --- | --- |
| Si | N/A | N/A | 8,000 |
| 5 | 40 | 1.35 | 15,000 |
| 5 | 45 | 1.70 | 26,000 |
| 5 | 50 | 1.98 | 31,000 |
| 23 | 50 | 1.54 | 22,000 |
| 45 | 50 | 1.25 | 15,000 |

1. **Confocal Laser Scanning Microscopy**

The following procedure was performed on 3D confocal microscope images using Gwyddion:

- 1. The data was levelled by mean plane subtraction.
  2. The outliers were masked.
  3. Performed a polynomial background subtraction.
  4. Applied gaussian filtering excluding the mask.
  5. The data was mean trimmed for 5% of the upper and lower values symmetrically and compared to untrimmed analysis (*Table S2*).
  6. 2D surface roughness parameters were evaluated, where *S_q_* is the root mean square roughness and *S_a_* is the mean roughness.
  7. 1D line roughness analysis provided the root mean square roughness (*R_q_*) / waviness (*W_q_*) and average roughness (*R_a_*) / waviness (*W_a_*).

***Table S2.*** *Roughness parameters for the silica substrate and rubber films processed using Gwyddion.*

|  | **Trimmed Parameters (nm)** | | | | | | **Untrimmed Parameters (nm)** | | | | | |
| --- | --- | --- | --- | --- | --- | --- | --- | --- | --- | --- | --- | --- |
| **Sample** | ***S_q_*** | ***S_a_*** | ***R_q_*** | ***R_a_*** | ***W_q_*** | ***W_a_*** | ***S_q_*** | ***S_a_*** | ***R_q_*** | ***R_a_*** | ***W_q_*** | ***W_a_*** |
| Si | 18.3 | 4.0 | 3.5 | 2.0 | 1.9 | 1.5 | 20.8 | 9.0 | 3.5 | 2.1 | 7.5 | 6.3 |
| 5 wt% | 30.0 | 5.9 | 3.6 | 2.4 | 6.9 | 4.7 | 74.3 | 52.5 | 4.7 | 3.1 | 67.5 | 54.0 |
| 23 wt% | 25.5 | 10.1 | 5.8 | 4.5 | 8.8 | 6.4 | 80.4 | 58.2 | 6.3 | 5.5 | 68.2 | 54.9 |
| 45 wt% | 22.3 | 14.0 | 6.5 | 4.8 | 16.4 | 12.1 | 102.2 | 78.8 | 9.5 | 6.5 | 85.2 | 65.4 |


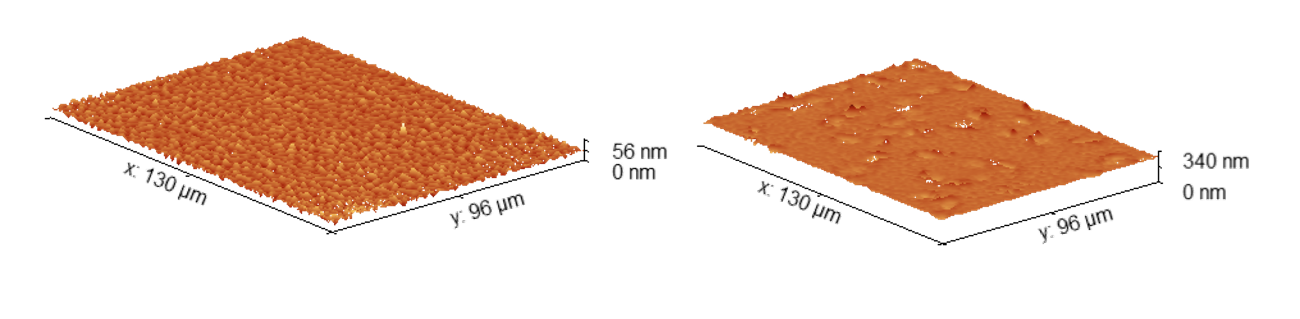


***Figure S4.*** *Example Gwyddion 3D images with 5% mean trimming: (Left) silica lens substrate (Right) 5 wt% rubber film.*

1. **Indentation Depth Calculations**

Two crossed cylinders (*i.e.* the silica lens surface positioned orthogonally to the ice surface) were treated as a ball-on-flat geometry to calculate the indentation depth (*d*) as shown by

𝑎!

𝑑 = 2𝐶_!_𝑅

where *a* is the contact radius, and *R* is the radius of the indenter and *C* is the degree of piling-up or sinking-in of the deformed surface.

Optical microscope images (*cf.* Figure S3) show the contact area to be elliptical in shape, and so the indentation depth equation would underestimate the value due to the difference in the radius of curvature between the surfaces ice (*R*_ice_) and silica (*R_si_*). As a result, the effective contact radius (*a_eff_*) for the elliptical contact and *R_ice_* were estimated using 𝐴 = 𝜋𝑎"##! , and +$%&’"./’(( *#*# "++%,-""++%,-" = *00!$!"#, respectively. An effective radius of curvature, *R*eff,

was estimated using ^1^ = ^1^ + ^1^ . Finally, *R*_eff,_ and *a_eff_* were substituted into the

0#%% 0!"# 0$!

indentation depth equation. The following Table S3 shows the indentation depth calculated with *R*_ice_ contributions or without, and assuming *C^2^* = 0.5 (elastic deformation):

***Table S3.*** *Calculated indentation depths for each sample at the ice interface.*

|  |  | **Sample indentation depth (nm)** | |  |
| --- | --- | --- | --- | --- |
| ***R* parameter used** | **Si** | **5 wt%** | **23 wt%** | **45 wt%** |
| *Reff* | 380 | 1400 | 940 | 710 |
| *R_si_* | 130 | 480 | 320 | 240 |

**4 Resonance Shear Measurements**

The styrene content study was averaged across three experiments (Ex) for each sample: ***4.1 Silica - Ice***


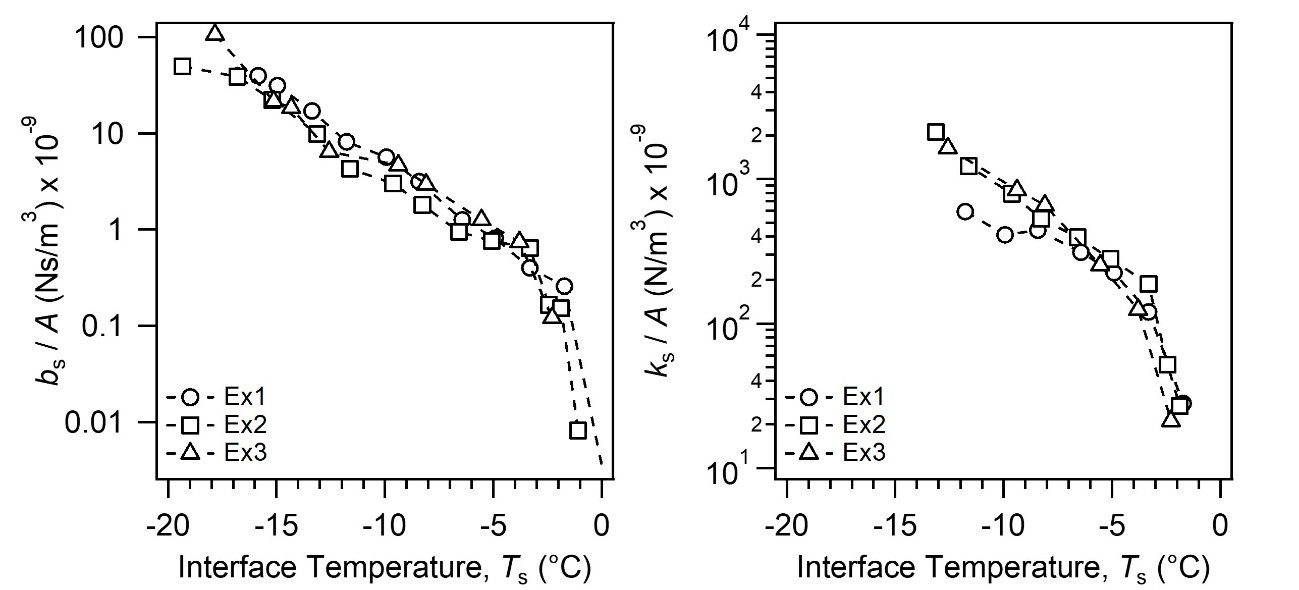


***Figure S5.*** *Viscoelastic parameters* (*b_s_*:left, *and k_s_*:right) *normalised to apparent contact area* (*A*) *vs temperature for the silica-ice interface.*

# 4.2 45 wt% Styrene - Ice


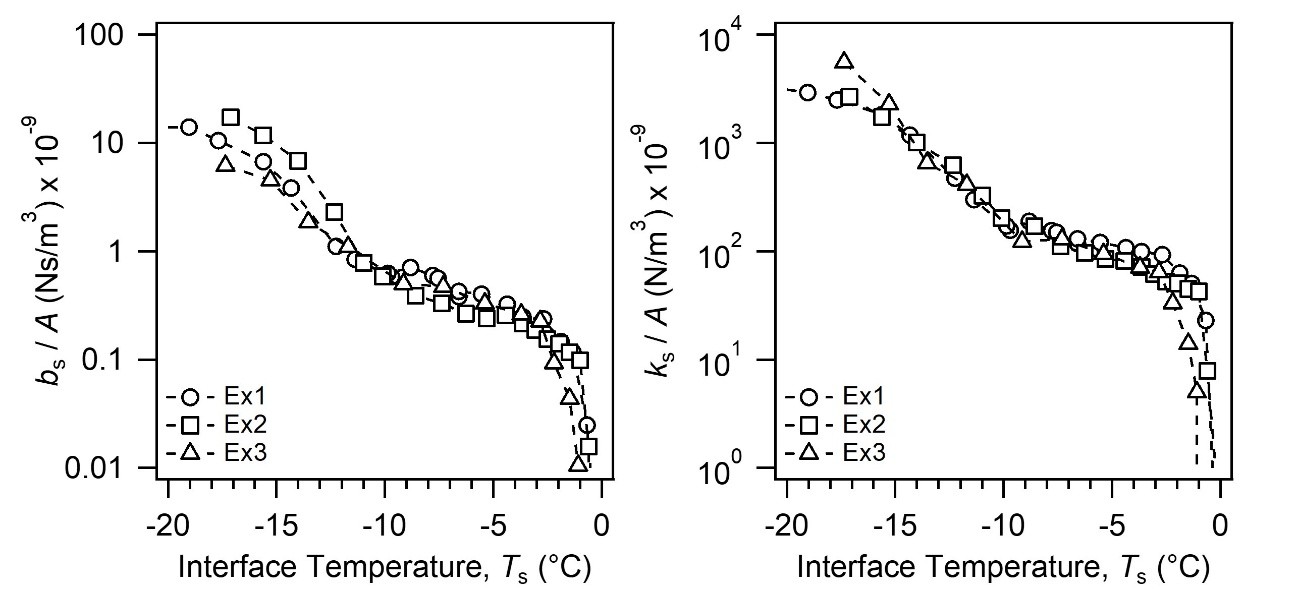


***Figure S6.*** *Viscoelastic parameters* (*b_s_*:left, *and k_s_*:right)  *normalised to apparent contact area vs temperature for the 45 wt% Styrene -ice interface.*

# 4.3 23 wt% Styrene - Ice


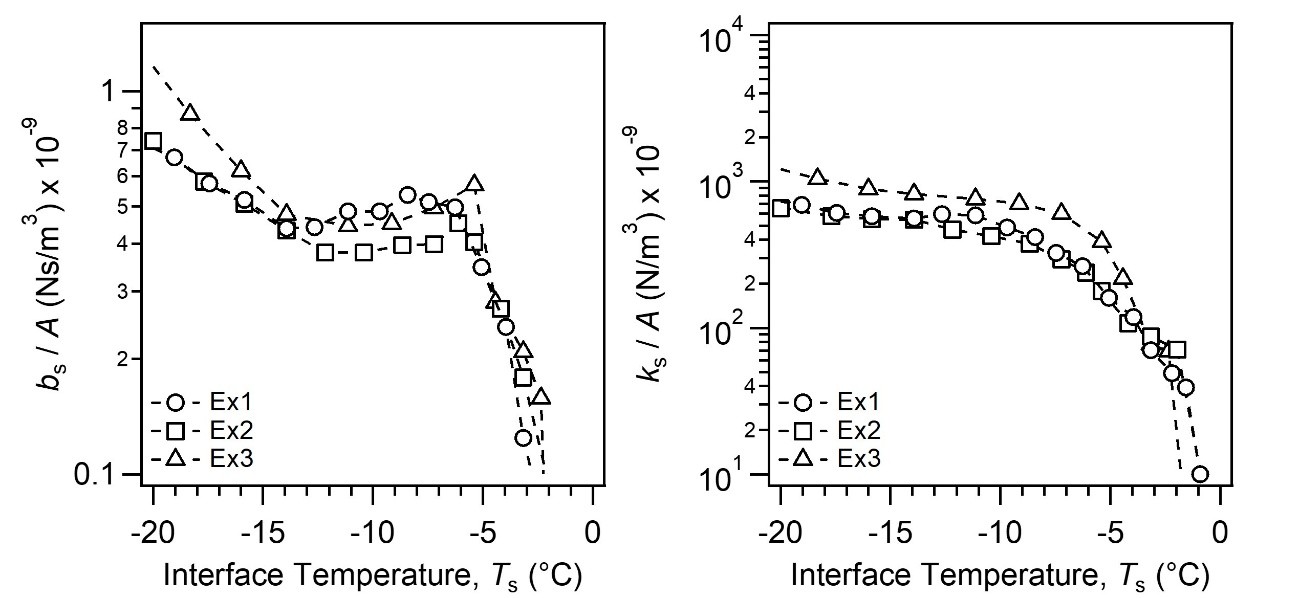


***Figure S7.*** *Viscoelastic parameters* (*b_s_*:left, *and k_s_*:right) *normalised to apparent contact area vs temperature for the 23 wt% Styrene -ice interface.*

# 4.4 5 wt% Styrene - Ice


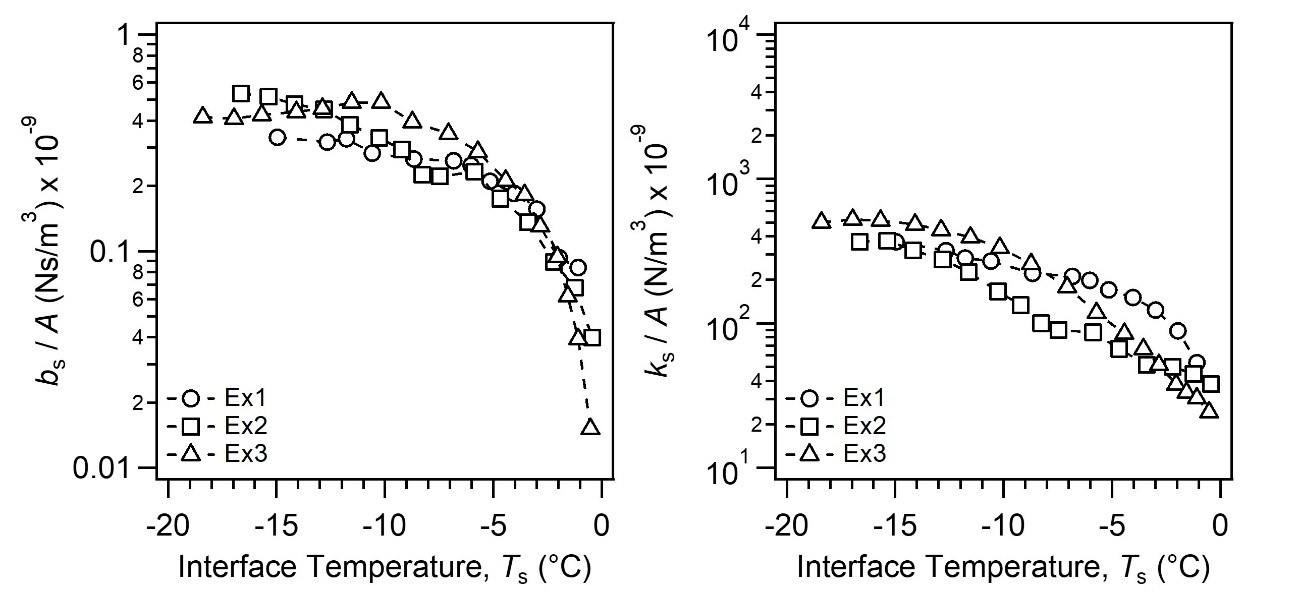


***Figure S8.*** *Viscoelastic parameters*(*b_s_*:left, *and k_s_*:right) *normalised to apparent contact area vs temperature for the 5 wt% Styrene -ice interface.*
